# Anti-biofilm activity of chlorhexidine digluconate against *Candida albicans* vaginal isolates

**DOI:** 10.1371/journal.pone.0238428

**Published:** 2020-09-17

**Authors:** Cathrin Alvendal, Soumitra Mohanty, Nina Bohm-Starke, Annelie Brauner

**Affiliations:** 1 Division of Obstetrics and Gynecology, Department of Clinical Sciences, Karolinska Institutet, Danderyd Hospital, Stockholm, Sweden; 2 Division of Clinical Microbiology, Department of Microbiology, Tumor and Cell Biology, Karolinska Institutet and Karolinska University Hospital, Stockholm, Sweden; University of Minnesota, UNITED STATES

## Abstract

**Objectives:**

Recurrent vulvovaginal candidiasis (RVVC) causes significant morbidity. *Candida albicans* is the main pathogen associated with both sporadic and recurrent candidiasis. Due to unsatisfactory treatment effect, the impact of chlorhexidine digluconate and fluconazole alone or in combination on *C*. *albicans* and biofilm was investigated.

**Methods:**

Vaginal *C*. *albicans* isolates from 18 patients with recurrent candidiasis and commensals from 19 asymptomatic women were isolated by culture. Crystal violet, XTT and colony forming unit assay were used to analyze the effect of chlorhexidine digluconate and fluconazole on growth of C. albicans, formation of new and already established, mature, biofilm.

**Results:**

Fluconazole reduced the growth of planktonic *C*. *albicans*. However, in established biofilm, fluconazole had no effect on the candida cells and was not able to disperse and reduce the biofilm. By contrast, chlorhexidine digluconate had a direct killing effect on *C*. *albicans* grown both planktonically and in biofilm. Chlorhexidine digluconate also dispersed mature biofilm and inhibited formation of new biofilm. No major differences were observed between commensal isolates and candida causing recurrent vulvovaginitis with respect to biofilm or growth after chlorhexidine digluconate treatment.

**Conclusion:**

Biofilm is a problem in patients with recurrent vulvovaginal candidiasis reducing the effect of antifungal treatment. Development of new treatment strategies are urgently needed to decrease the recurrences. In already established biofilm, chlorhexidine digluconate dispersed the biofilm and was more effective in eradicating candida compared to fluconazole. Future treatment strategy may thus be a combination of chlorhexidine digluconate and fluconazole and prophylactic use of chlorhexidine digluconate to prevent biofilm formation and restrict infections.

## 1. Introduction

Vulvovaginal candidiasis (VVC) is a common infection and affects 70–75% of all women[1]. Many women will experience relapses of the infection and as many as 5–8% suffer from recurrent vulvovaginal candidiasis (RVVC), defined as three or more infections per year [1, 2]. Well known risk factors for sporadic VVC are diabetes mellitus, pregnancy and use of antibiotics [1]. In idiopathic RVVC, however, triggering factors are not obvious and many other mechanisms such as virulence factors of candida species, ineffective immune response and genetic factors have been discussed [3]. RVVC causes significant morbidity in affected women with a profound effect on the quality of life and increased risk to develop depression, anxiety and provoked vestibulodynia [3, 4].

*Candida albicans* colonizes skin and mucosal membranes and 85–95% of yeast strains isolated from the vagina belong to *C*. *albicans* [1]. However, *C*. *albicans* can cause not only local, but also severe systemic infections like candidemia with a mortality rate of 40% [5, 6]. Yeast blastospores is the phenotypic form responsible for vaginal transmission and asymptomatic colonization of the vagina [1]. Germinated yeast, which have produced hyphae, are found in symptomatic vaginal infections [1, 7]. Treatment of RVVC is complex but control of the infection is often achievable with the use of fluconazole (FLZ) suppressive therapy for a period of six months, but cure of RVVC remains elusive [8].

FLZ is fungistatic and inhibits the synthesis of ergosterol, an important component of the candida cell membrane [9]. Due to the pharmacodynamics of fluconazole with a long half-life time of 25h, concentrations above the minimal inhibitory concentration (MIC) that inhibits the growth of 90% of candida species are achieved for 72 h in vaginal secretion. Treatment with weekly administration is considered appropriate [8, 10]. The long-term therapy is proven non-toxic with minimal side effects [8, 11]. However, penetration of FLZ into the candida biofilm is poor leading to a low drug concentration and thereby a possible development of resistance has been suggested [12], although resistance is still considered uncommon [13].

Biofilm is a survival mechanism used by both pathogenic and commensal microorganisms. It can be formed on abiotic and biotic surfaces [14, 15], and are considered to contribute to about 80% of microbial infections in the body [16]. Formation of the *C*. *albicans* biofilm is induced by the adherence to the epithelial cells with help of adhesins of the fungal cell surface [17]. The biofilm of *C*. *albicans* is a complex three-dimensional structure containing an exopolymer matrix and a mixture of yeast, pseudohyphae and hyphae [6, 18] which protects the microorganisms from the immune system and antifungal therapy [19, 20]. Some of the candida cells within the biofilm are in a non-growth state and target molecules are therefore not available to antifungals [20–23]. Current antifungals have limited effect reaching candida cells inside the biofilms [24], therefore new treatment strategies to disperse the biofilm as well as preventing new biofilm formation are needed [17].

A combination of genetic factors, altered local immune response in the host, vaginal environment and candida virulence factors might enhance the risk of fungal infection [3] and thereby also affect biofilm formation [17]. Transition of candida to hyphal forms is accompanied by the assembly of biofilm and triggers tissue damage and activation of host innate immune response with production of inflammatory mediators, recruitment of neutrophils and resulting in symptomatic infection [25]

The antifungal effect of chlorhexidine digluconate (CHG) has been demonstrated in clinical trials and has been used successfully in a regimen for the treatment of oral candidosis in otherwise healthy individuals [26]. It has been shown that chlorhexidine solutions can diminish denture biofilm [27], but studies on vaginal use are rare. A study by Shubair et al, showed no profound changes in the vaginal flora after 7 days of vaginal treatment with CHG [28].

In RVVC, it has been shown that most of the recurrences are caused by the same candida strains despite antifungal therapy [29]. We speculate that *C*. *albicans* can remain within the biofilm and when released cause recurrent infection. To eradicate the biofilm and *C*. *albicans*, we investigated the effect of CHG, alone and in combination with FLZ, both on already established biofilm and on the possibility to prevent the formation of new biofilm. The anti- candida effect of CHG and/or FLZ was also analyzed in RVVC and commensal isolates.

## 2. Material and methods

### Participant and samples

*Candida albicans* isolated in vaginal swabs were obtained from 18 women, median age 31 years (range 20–39), with RVVC. All women had a history of 3–4 candida infections during the last year and were suffering from an acute episode at the time of the study. The patients had no known predisposing underlying conditions including pregnancy, antibiotic treatment, diabetes or immunosuppression. As controls served *C*. *albicans* isolates from 19 asymptomatic women, referred to as commensals and included for control purposes. The patients were recruited at the outpatient vulvar clinic at Danderyd Hospital, Stockholm, Sweden, during a 2 years’ period.

None of the patients had received any antibacterial or antifungal treatment prior to the study. After examination and culture sampling they received antifungal treatment with oral fluconazole 50 mg per day for one week, followed by oral fluconazole 150 mg per week for another five consecutive weeks. After six weeks, when the treatment was completed, they came for a follow-up visit and new cultures were all negative for *C*. *albicans*. Ethical permission for the study was given by the Regional Ethics Committee in Stockholm, and informed consent were obtained from all patients.

### Isolation and identification of *Candida albicans*

Vaginal swabs were cultured on CHROMagar^TM^ and *C*. *albicans* were identified by typical colony pigmentation and latex surface antigen agglutination (Bichro-Dubli). Isolates were kept frozen at -80°C. For experiments, if not stated otherwise, isolates were cultured on Sabouraud agar for 1 day at 37°C and then in yeast peptone dextrose (YPD) medium overnight at 30°C. This culture was diluted 1:100 in fresh medium and grown for another 3 h at 30°C to reach the logarithmic growth phase. Under these conditions, *C*. *albicans* grew as yeast cell.

### Minimum Inhibitory Concentration (MIC) assay

The minimum inhibitory concentrations (MIC) for chlorhexidine digluconate (CHG) and fluconazole (FLZ) was determined for each of the commensal and RVVC strains using a broth microdilution method in 96-well polystyrene microtiter plate according to EUCAST guidelines [30]. The stock solution of twofold serial dilutions of FLZ and tenfold serial dilutions of CHG in MOPS RPMI buffer with the final concentrations ranged between 64 to 0.125 μg/ml for FLZ and 0.02% to 0.00002% for CHG. *C*. *albicans* were grown for 36h at 35°C on Sabouraud agar plates and suspended in sterile water and turbidity was measured spectrophotometrically at 530 nm. Final candida solution was 1–5 x 10^6^ CFU /mL. 100μl of inoculum was added to the microtiter plate, incubated at 35°C for 24h, optical density (OD) was measured at 450nm. *C*. *parapsilosis* and *C*. *krusei* were used as control strains (Table 1).

**Table 1:** 

RVVC			Commensals		
Strain number	FLZ MIC (μg/ml)	CHG MIC (%)	Strain number	FLZ MIC (μg/ml)	CHG MIC (%)
SV 6	0.250	0.0002	Comm. 3	0.250	0.002
SV 9	0.125	0.0002	Comm. 4	0.250	0.002
SV 15	0.250	0.0002	Comm. 5	0.125	0.0002
SV 19	0.125	0.0002	Comm. 6	0.125	0.002
SV 24	0.125	0.0002	Comm. 8	0.250	0.0002
SV 27	0.250	0.0002	Comm. 9	0.125	0.002
SV 28	0.250	0.0002	Comm. 10	0.125	0.002
SV 32	0.250	0.0002	Comm. 12	0.125	0.002
SV 34	0.250	0.0002	Comm. 13	0.125	0.002
SV 35	0.250	0.0002	Comm. 14	0.125	0.002
SV 36	0.125	0.0002	Comm. 15	0.125	0.002
SV 38	0.125	0.0002	Comm. 16	0.250	0.002
SV 41	0.250	0.0002	Comm. 17	0.250	0.002
SV 42	0.125	0.0002	Comm. 18	0.250	0.0002
SV 43	0.125	0.0002	Comm. 19	0.250	0.0002
SV 45	0.250	0.0002	Comm. 20	0.125	0.0002
SV 48	0.250	0.0002	Comm. 21	0.250	0.0002
SV 57	0.125	0.0002	Comm. 22	0.125	0.0002
			Comm. 23	0.125	0.0002
*C*.*parapsiolis*	2	0.00002		2	0.00002
*C*.*krusei*	32	0.00002		32	0.00002

*C*. *krusei* is positive control and *C*. *parapsilosis* is negative control.

### *C*. *albicans* biofilm measured with the microtiter method

To measure the ability of *C*. *albicans* to adhere and form biofilm, the crystal violet assay in polystyrene microtiter plates (Costar Corning) was performed. *C*. *albicans* in concentration 5 x 10^6^, were grown for 48 h in 200 μl YPD (BD, Sparks) at 30°C without shaking. Biofilm was washed with 1× PBS and stained with 200μl of crystal violet (0.3%) for 10 min in room temperature. Unbound dye was removed, stained biofilms were washed with water. The dye was solubilized with 220 μl of 80% ethanol and 20% acetone and the OD was measured at 570 nm. Blank was subtracted from all test strains.

### Microtiter method to measure *C*. *albicans* metabolic active cells

The effect of CHG and FLZ treatment on *C*. *albicans* metabolic activity and proliferation was determined using an XTT assay. Newly formed and mature biofilms of commensals and RVVC isolates were treated with different concentrations of CHG (S1 Fig) or FLZ for 48 h or 72 h. Samples were then incubated with 200μl of 20% solution of 1 mg/ml XTT (Sigma) and 12.5 mM menadione (Sigma) in YPD for 4 h. The conversion of tetrazolium salt XTT to a colored formazan derivative was measured at 450 nm in a 96-well plate. Non-treated controls were maintained throughout the cell viability assay. Blank was subtracted from all test strains.

### Evaluation of chlorhexidine digluconate and fluconazole on already established, mature, biofilm and on the formation of new biofilm

The effect of chlorhexidine digluconate (0.02%, GlaxoSmithKline) or fluconazole (4 μg/ml, Sigma) alone or in combination was evaluated on already established, mature biofilm, as well as on the formation of new biofilm. We first analyzed the effect on established biofilm and allowed biofilm to establish for 48 h, before CHG, FLZ or a combination was added for 24 h. Next, we investigated the effect on formation of new biofilm. This time only CHG was added already from the beginning and biofilm was evaluated after 48 h as described above. The OD of the chlorhexidine digluconate treated group with or without fluconazole was compared with a control group treated with medium.

### Enumeration of *Candida albicans* in mature and newly formed biofilm

*C*. *albicans* were enumerated in mature and newly formed biofilm. Mature biofilm was exposed to the effect of CHG, FLZ and the combination of CHG and FLZ or medium alone. Newly formed biofilm was exposed only to CHG alone and as control medium. To investigate if the tested drugs had impact on the candida growth, the number of planktonic cells as well as cells within the biofilm were analyzed. Planktonic candida cells detected in the medium were determined by viable count after serial dilutions in PBS. To assess the number of candida in the already established, mature, biofilm, the biofilm was first washed twice with PBS and then scraped from the microtiter plate in 200 μl PBS, and serially diluted before viable count was performed. Dilutions were plated on blood agar plates followed by 36 h incubation in 37°C and results from viable cells exposed to test drugs or medium alone were calculated.

### Effect of chlorhexidine digluconate on *C*. *albicans* hyphae

For newly formed biofilm *C*. *albicans* were cultured together with CHG in YPD broth at 30°C and 35°C for 48h. For established, mature biofilm, *C*. *albicans* were allowed to form biofilm for 48h. After that CHG was added to the mature biofilm for another 24h. 10μl of *C*. *albicans* were smeared on a glass slide, placed on 55°C for 15min. Blankophore p, was used to stain the glucan and chitin of the *C*. *albicans* cell wall. Slides were visualized in 20× objective under UV Olympus microscope.

### Statistics

Data were analyzed using IBM SPSS Statistics for Windows (Version 22) and GraphPad Prism 5.00 for Windows (GraphPad Software, Inc.). The formation of untreated and treated biofilms (CHG/FLZ) in both RVVC and commensals were analyzed with one-way ANOVA and Bonferroni's Multiple Comparison Test. The same test was used for analyzing cell growth by viable count (CFU/ml) of planktonic cells and candida cells within mature biofilm. Significant level was set to p <0.05.

## 3. Results

### Fluconazole has limited effect on RVVC and commensal strains in already established biofilm

**Fig 1:**
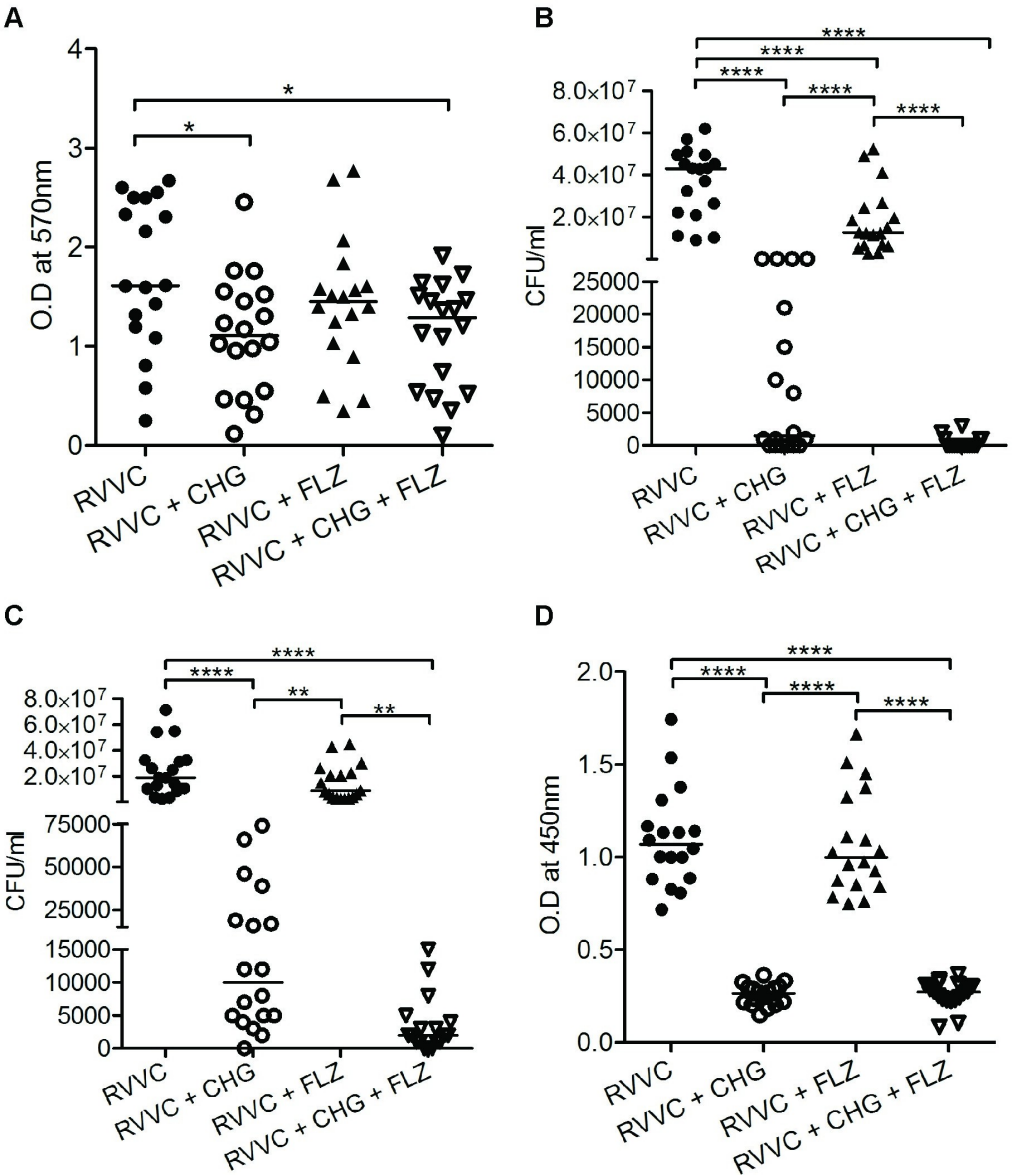
Chlorhexidine digluconate reduces already established biofilm and growth of *C*. *albicans* isolates from patients with RVVC. The effect of chlorhexidine digluconate (CHG) 0.02% and fluconazole (FLZ) 4 μg/ml and the combination CHG + FLZ compared to untreated *C*. *albicans* was investigated on RVVC strains (n = 18). For RVVC, density of established, mature, biofilm, measured with crystal violet staining **(A),** viable count of planktonic *C*. *albicans* (**B)** and of *C*. *albicans* within mature biofilm (**C)**. Metabolic active cells within the mature biofilm, measured with XTT assay **(D).** Individual and median values are presented, depicted as optical density (OD) at 570 nm after dissolution of crystal violet, and at 450 nm for soluble product of formazan crystal after reduction of XTT. * p<0.05, ** p<0.01, *** p <0.001 and **** p <0.0001.

Biofilm is well-known for its ability to prevent antimicrobial drugs and innate immunity to reach their targets. Women with RVVC can harbor *C*. *albicans* in already established biofilm, from where isolates can be expelled and cause new infections. Therefore, we investigated the effect of FLZ, the most commonly used treatment for RVVC during these conditions. To mimic the clinical situation, we analyzed the killing effect on planktonic as well as *C*. *albicans* within biofilm, Fig 1A–1D. We observed a significant decrease of planktonic candida cells after FLZ treatment (p<0.0001), Fig 1B. However, no effect was observed on candida growing within the biofilm, Fig 1C. Likewise, FLZ did not affect the biofilm *per se*, Fig 1A nor the metabolic activity of RVVC within the biofilm, Fig 1D.

**Fig 2:**
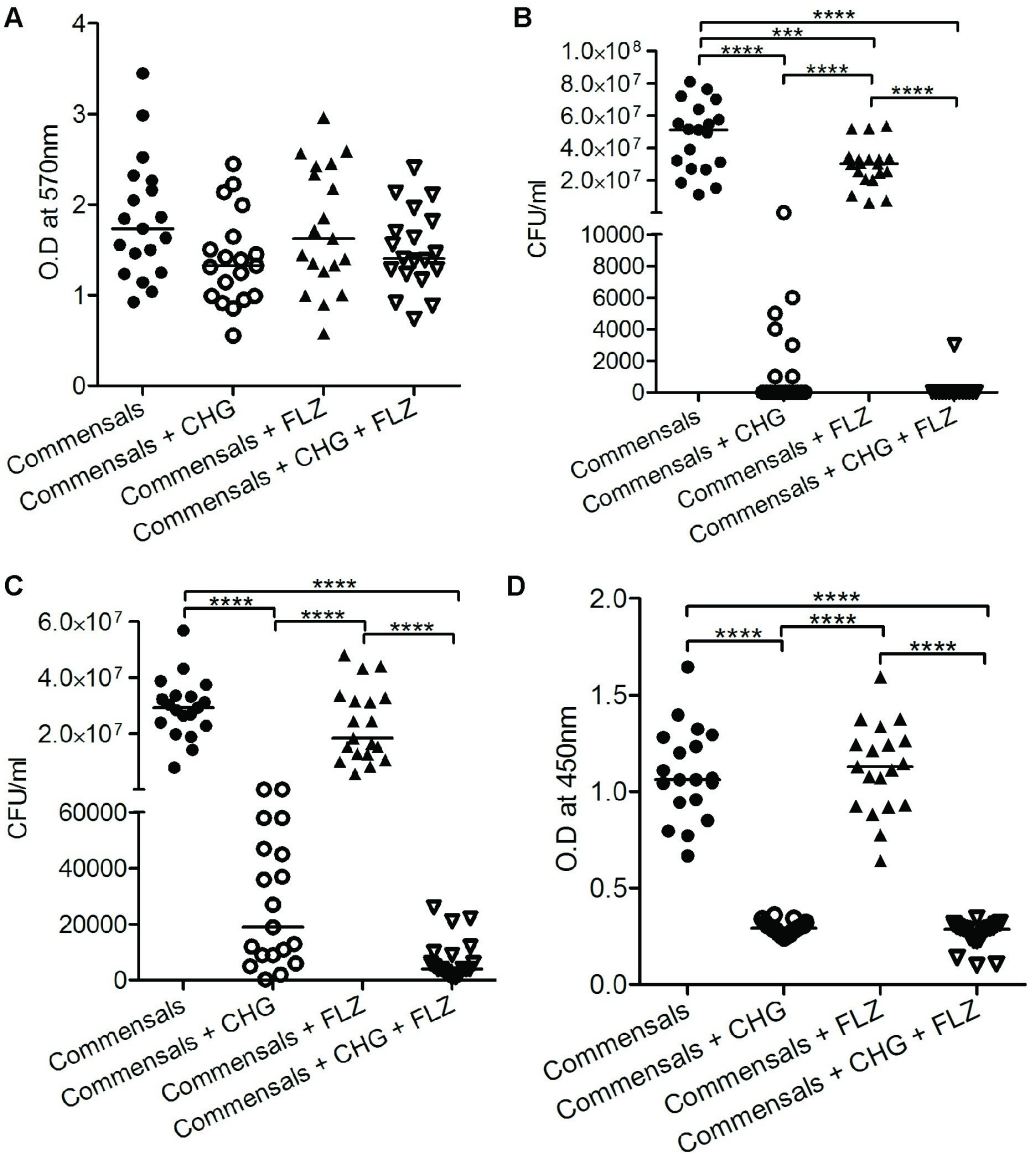
Chlorhexidine digluconate reduces already established biofilm and growth of *C*. *albicans* isolates from asymptomatic women. The effect of chlorhexidine digluconate (CHG) 0.02% and fluconazole (FLZ) 4 μg/ml and the combination CHG + FLZ compared to untreated *C*. *albicans* was investigated on commensals strains (n = 19). The density of established, mature, biofilm, measured with crystal violet staining **(A),** viable count of planktonic *C*. *albicans* (**B)** and of *C*. *albicans* within mature biofilm (**C)**. Metabolic active cells within the mature biofilm, measured with XTT assay **(D).** Individual and median values are presented, depicted as optical density (OD) at 570 nm after dissolution of crystal violet, and at 450 nm for soluble product of formazan crystal after reduction of XTT. * p<0.05, ** p<0.01, *** p <0.001 and **** p <0.0001.

**Fig 3:**
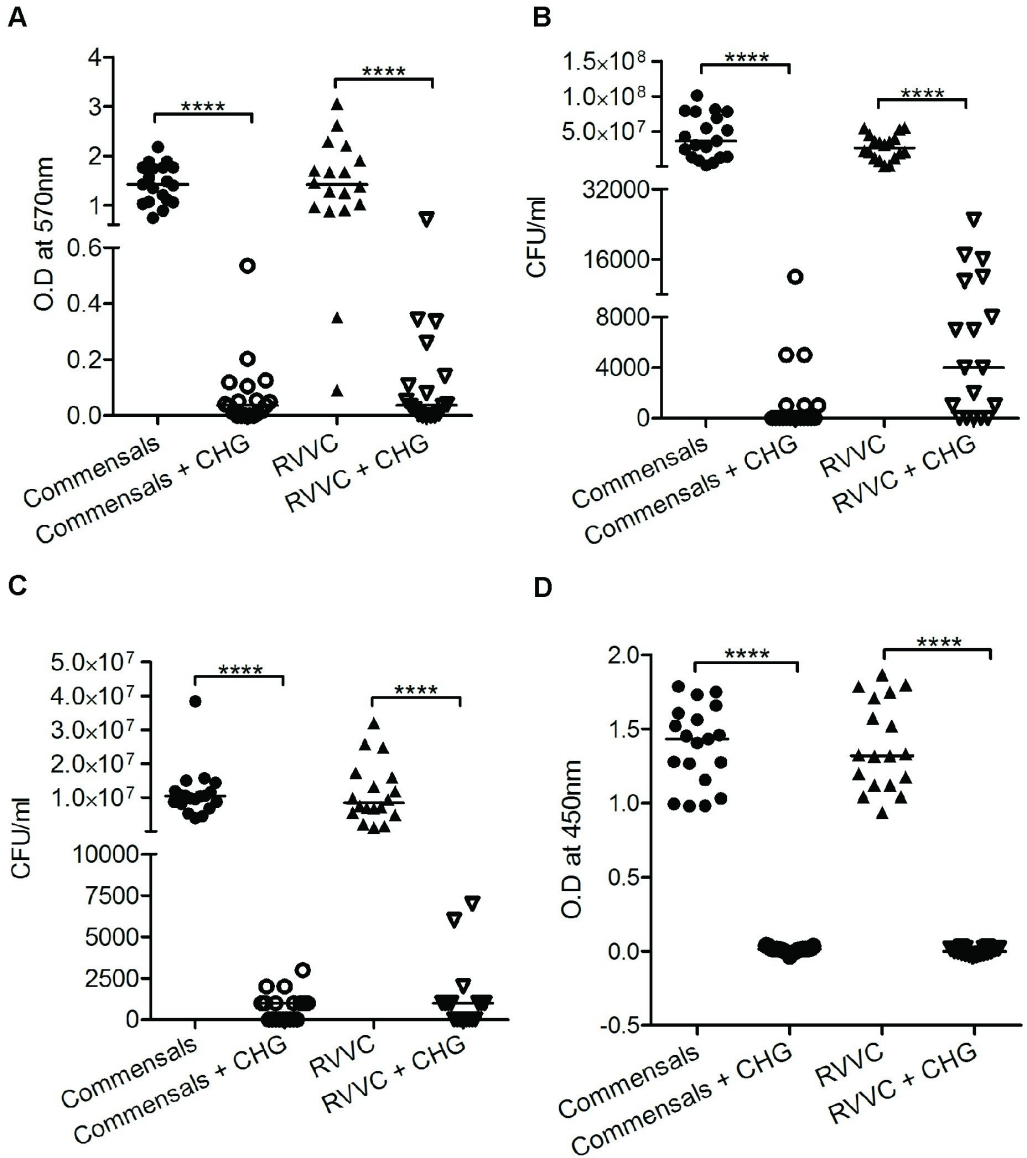
Chlorhexidine digluconate inhibits the formation of new biofilm and growth of *C*. *albicans*. The effect of chlorhexidine digluconate 0.02% was investigated on formation of new *C*. *albicans* biofilm using crystal violet assay (**A**). Viable count was shown for planktonic *C*. *albicans* (**B)** and of *C*. *albicans* in newly formed biofilm (**C)**. Metabolic active cells within the mature biofilm, measured with XTT assay **(D).** Untreated strains served as controls. Individual and median values are presented, depicted as optical density (OD) at 570 nm after dissolution of crystal violet, and at 450 nm for soluble product of formazan crystal after reduction of XTT. **** p <0.0001.

The commensal isolates behaved similar to the RVVC strains when exposed to FLZ and CHG. However, CHG alone or in combination with FLZ showed a tendency to reduce mature biofilm, but did not reach significance, Fig 2A, which was contrary to the RVVC strains, Fig 1A. CHG alone and in combination with FLZ were capable of inhibiting metabolically active commensal candida growth both in the planktonic phase and within already established biofilm Fig 2B–2D. However, CHG effectively and equally prevented formation of new biofilm of RVVC and commensal isolates, Fig 3A.

### Chlorhexidine digluconate disperses established biofilm of RVVC strains and inhibits candida growth

The relatively poor effect of FLZ on biofilm embedded candida and on biofilm *per se*, prompted us to analyze alternative treatment strategies. Several studies have shown the antimicrobial effect of CHG [26, 31]. In line with these studies, *C*. *albicans* growth both in the planktonic state and within biofilm, was significantly reduced by CHG treatment, Fig 1B and 1C. The combination of CHG and FLZ further reduced the candida growth within mature biofilm, compared to CHG alone, although it did not reach significance. Contrary to FLZ alone, CHG either as single treatment or in combination with FLZ significantly reduced the mature biofilm, Fig 1A and 1D. The fact that we only observed a limited reduction of biofilm is particularly interesting taking the pronounced inhibition of candida growth into consideration.

Dose dependent effect of CHG on mature *C*. *albicans* biofilm was tested on RVVC and commensal strains. We observed a similar and significant effect only of 0.02% CHG, which consequently was used in all the experiments, S1 Fig.

### Chlorhexidine digluconate inhibits formation of new biofilm in RVVC and commensal strains

*C*. *albicans* persisting in the vagina between overt infections, are regarded as a precondition for RVVC. Planktonic candida as well as candida released from biofilm candida can cause infections and also form new protective biofilm. To eradicate these candida cells and prevent biofilm formation is therefore crucial to avoid new infection episodes. Based on the effect of CHG on mature biofilm, we sought to investigate if CHG also could inhibit formation of new biofilm and *C*. *albicans* growth. We observed that CHG was able to prevent the formation of new biofilm and inhibit candida growth in planktonic and growing biofilm, both in the RVVC and commensal strains, Fig 3A–3D.

### Chlorhexidine digluconate inhibits the hyphal growth of *C*. *albicans*

**Fig 4:**
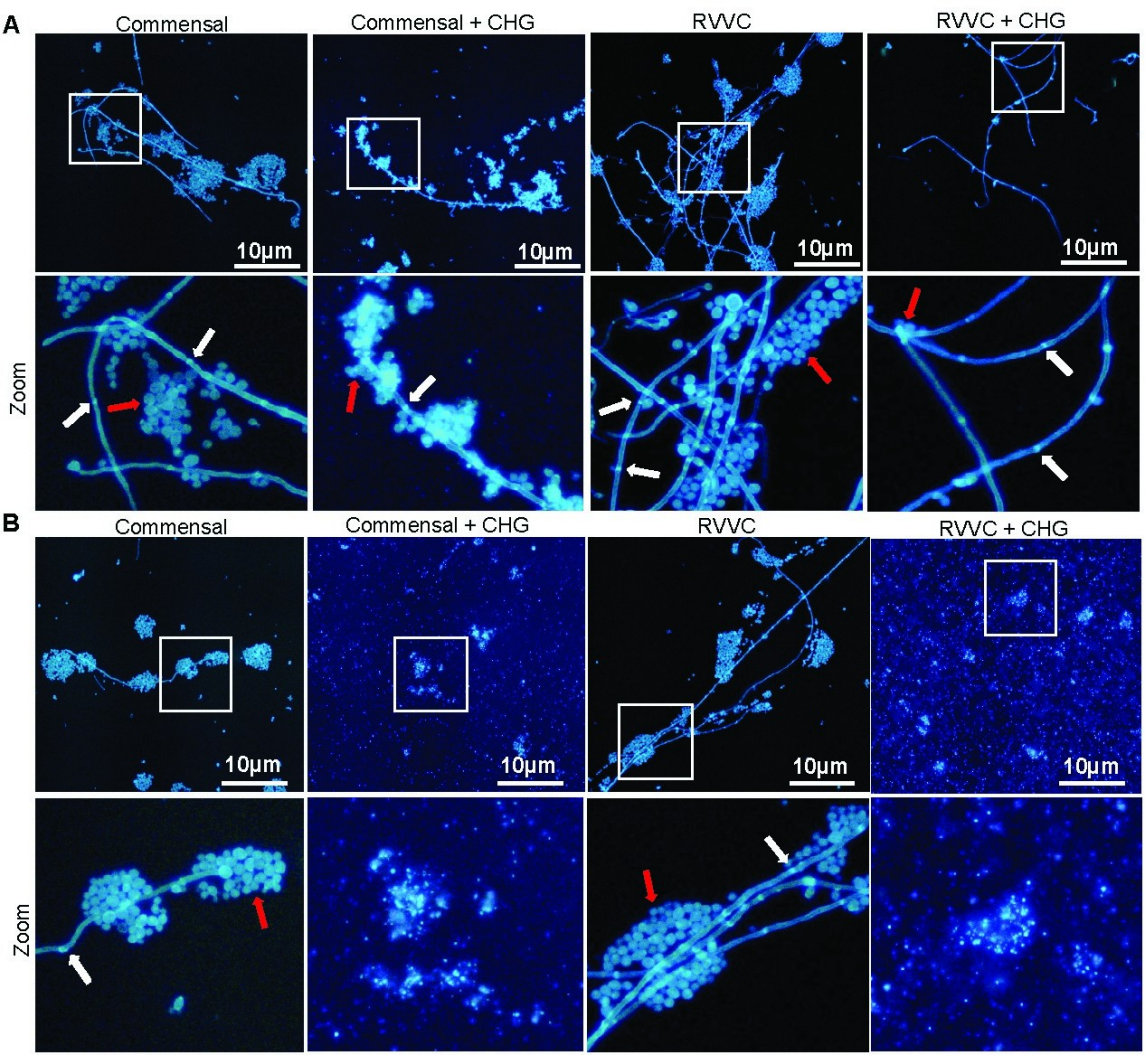
Chlorhexidine digluconate inhibits the hyphal growth of *C*. *albicans*. *C*. *albicans* obtained from patients with RVVC (n = 5) and from asymptomatic women, commensal strains (n = 5) were treated with chlorhexidine digluconate 0.02%. Imaging of hyphae from mature biofilm after 48h and another 24h with CHG treatment (**A).** Newly formed biofilm was evaluated after 48h growth together with CHG **(B).** Staining was performed using blankophore p, images were captured using 20× objective under UV in Olympus microscope. Representative images are shown. Marked areas were magnified, lower panel. Fungal septa are marked with white arrows and budding cells with red arrows.

Mature biofilms are complex mixes of yeast, pseudohyphal and hyphal cells. To confirm hyphal growth in YPD medium, we investigated the effect of CHG upon fungal hyphal growth in RVVC and commensal *C*. *albicans* isolates. In line with our results from CFU in mature biofilm (Figs 1C and 2C), 24h CHG treatment alone significantly disrupted the hyphal network in mature biofilm of both RVVC and commensals irrespectively of temperature condition. However, in untreated *C*. *albicans* grown at 30° C, a few strains formed hyphae while others did not (S2A and S2B Fig). Budding cells and hyphal septum were compromised after CHG treatment, Fig 4A. Similarly, in newly formed biofilm, CHG did not allow the formation of fungal hyphal growth when compared to untreated *C*. *albicans*. Both RVVC and commensals cells were completely destroyed with absence of budding cells, Fig 4B.

### Minimum Inhibitory Concentration (MIC) of Fluconazole and chlorhexidine digluconate on commensals and RVVC

The MIC values for fluconazole for the RVVC strains were median 0.25 mg/ml (IQR 0.125–0.25) and 0.125 mg/ml (IQR 0.125–0.25) for the commensal strains. For CHG, median MIC values for RVVC was 0.0002% (IQR 0.0002–0.0002) and for the commensals 0.002% (IQR 0.002–0.0002), Table 1. None of the differences in MIC values between RVVC and commensal strains were significant.

## 4. Discussion

Treatment of RVVC is difficult and challenging for both patients and physicians. In this study, we demonstrate that *C*. *albicans* from these women form biofilm and that fluconazole has no impact either on biofilm or on candida growth within already established biofilm. Therefore, alternative treatment strategies to disperse biofilm and make microorganisms accessible as well as prevention of new biofilm formation for women with RVVC is important.

Since recurrences of vulvovaginal candidiasis is a major clinical problem, we assume that biofilm plays a major role for the microorganisms to hide and persist, then disperse from the biofilm and eventually cause recurrent infections. Therefore, it is interesting to notice that we did not observe any difference between RVVC and commensal strains, with respect to biofilm formation. Nevertheless, we cannot rule out that commensal strains under other conditions may become pathogenic and cause infection. In a study by Swidsinsky *et al* [32] biofilm was not detected in vaginal tissue biopsies from patients with vulvovaginal candidiasis. However, it was recently argued by Noverr and Fidel (28) that the methods used to detect biofilm elements were not optimal and that the biofilm could have been dispersed during biopsy preparation [33].

Long term treatment with FLZ is considered first choice for RVVC [3], however, FLZ does not have the capability to disperse already existing mature biofilm. Moreover, we demonstrate that FLZ was not able to reduce candida growth within the biofilm in contrast to the significant reduction of planktonic cells. If our results are applicable in a clinical setting of VVC need to be confirmed. The effect from azoles on artificial surfaces might be different from the effect on the vaginal candidiasis. Although the widespread use of long-term treatment with FLZ, there is yet no evidence of increased resistance frequency [3, 34]. However, patients with confirmed resistant C *albicans* strains had previously been exposed to FLZ [13]. To minimize resistance development, other treatment options need to be considered. In this context, the importance of the results in this study is further emphasized.

Extracellular matrix formed by *C*. *albicans*, may also influence the effect of antifungal treatment [35]. Therefore, it cannot be ruled out, that the poor effect observed by FLZ may partly be due to the extracellular matrix.

CHG dispersed the mature biofilm and effectively inhibited formation of new biofilm. Treatment with CHG almost completely eradicated candida in the planktonic state. Another interesting observation was the pronounced reduction of candida growth within the mature biofilm, which did not correspond to the ability to disperse the biofilm. Our results indicate that even though significant amount of biofilm is present, CHG seems to penetrate the biofilm. One may speculate whether CHG changes the properties of the biofilm, making it possible for the molecules to reach and interact with the candida cell wall. This would further influence the killing effect by CHG leading to fewer candida cells and in turn less biofilm, which would further improve the treatment.

The yeast-to-hyphae transition contributes significantly in the progress of infection which can be life-threatening if they become systemic [36]. The hyphae penetrate through the mucosa and damage the epithelial cells causing the specific symptoms in vulvovaginal candidiasis [7, 15]. Hyphal growth is influenced by temperature and time [37]. In our study *C*.*albicans* was grown for prolonged time, at both low and high temperature. These findings highlight the importance of taking both growth time and temperature into consideration when evaluating fungal hyphal growth. Moreover, the effect of CHG on both mature and newly formed biofilm further reduces the ability of fugal hyphae in immune evasion, adhesion, and host cell damage. We have previously demonstrated that the antimicrobial peptide psoriasin binds to β-glucan, a basic component of the *C*. *albicans* cell wall, thereby inhibiting the adhesion of *C*. *albicans*, an early step of biofilm formation Similarly, hyphal filaments help in the adhesion and invasion of the host epithelium. In our previous report, we demonstrated that the antimicrobial peptide psoriasin acts as an anti-candida adhesin [38]. We therefore speculate that there could be a direct effect of psoriasin on the fungal hyphal network.

Based on our results, vaginal CHG might be an option to diminish recurrences in RVVC patients, especially when treatment fails. Vaginal CHG could be used for both therapeutic and prophylactic purposes. The number of detected *C*. *albicans* within established biofilm did not differ between CHG and the combination of CHG and FLZ samples. Even so, in symptomatic cases the combination with FLZ might clinically result in an enhanced eradication of candida. For prophylactic use, regular vaginal treatment with CHG could therefore inhibit biofilm formation and might consequently prevent recurrences. In a study by Salim *et al*, chlorhexidine was superior to FLZ in eradicating *C*. *albicans* isolates causing oral candidosis [39]. Antiseptic substances to treat and prevent vaginal candidiasis has only been investigated to a limited extent. In a study by Molteni et al, 87% of women treated with 0.5% chlorhexidine vaginal gel were clinically cured after one week’s treatment, but this regime needs to be further explored [40]. It has been shown that the use of 2% chlorhexidine gluconate as a vaginal preoperative preparation was not associated with increased vaginal irritation or allergic reactions [41] and that the tolerability in women using a vaginal 0.5% chlorhexidine gluconate gel for 7 days was good [40]. Moreover, the effect of the normal bacterial flora has to be taken into consideration as CHG has a general effect on microorganisms [31].

The major limitation of this study is the low number of *C*. *albicans* isolates and the results from CHG treatment on *C*. *albicans* and biofilm formation *in vitro* need to be confirmed in clinical trials. In addition, long term effect of vaginal CHG on the normal microbial flora and epithelial cells have to be evaluated.

## 5. Conclusion

Biofilm is a problem in patients with RVVC, reducing the effect of antifungal treatment. Development of new treatment strategies are therefore urgently needed to reduce the high rate of recurrences. In already established biofilm, chlorhexidine digluconate dispersed the biofilm and was more effective in eradicating candida compared to fluconazole. Future treatment strategy may thus be a combination of chlorhexidine digluconate and fluconazole to avoid relapses and prophylactic use of chlorhexidine digluconate to prevent biofilm formation and restrict infections.

## Supporting information

S1 Fig Dose response of chlorhexidine digluconate on already established biofilm of commensal *C*. *albicans* isolates from asymptomatic women. The effect of different concentration of chlorhexidine digluconate (CHG), 0.00002%- 0.02%, were compared to untreated *C*. *albicans* was investigated on both RVVC (n = 10) **(A)** and from asymptomatic women, commensals (n = 10) **(B)**. Metabolic active cells within the mature biofilm, analyzed with XTT assay and measured at 450nm. **** p <0.0001.
(TIF)

S2 Fig Chlorhexidine digluconate inhibits the hyphal growth of *C*. *albicans* at 30°C. *C*. *albicans* obtained from patients with RVVC (n = 10) and from asymptomatic women, commensals (n = 10) were treated with chlorhexidine digluconate 0.02%. Imaging of hyphae from mature biofilm after 48h and another 24h with CHG treatment at 30°C commensals **(A)** and RVVC **(B).** Staining was performed using blankophore p, images were captured using 20× objective under UV in Olympus microscope. 3 different representative images are shown.
(TIF)

## Abbreviations

VVC: vulvovaginal candidiasis
RVVC: recurrent vulvovaginal candidiasis
FLZ: fluconazole
CHG: chlorhexidine digluconate
