# Supplementary figures and images for: Anti-biofilm activity of chlorhexidine digluconate against Candida albicans vaginal isolates
Source: PLoS One. 2020 Sep 17;15(9):e0238428. doi: 10.1371/journal.pone.0238428 (PMC7498037; doi:10.1371/journal.pone.0238428)

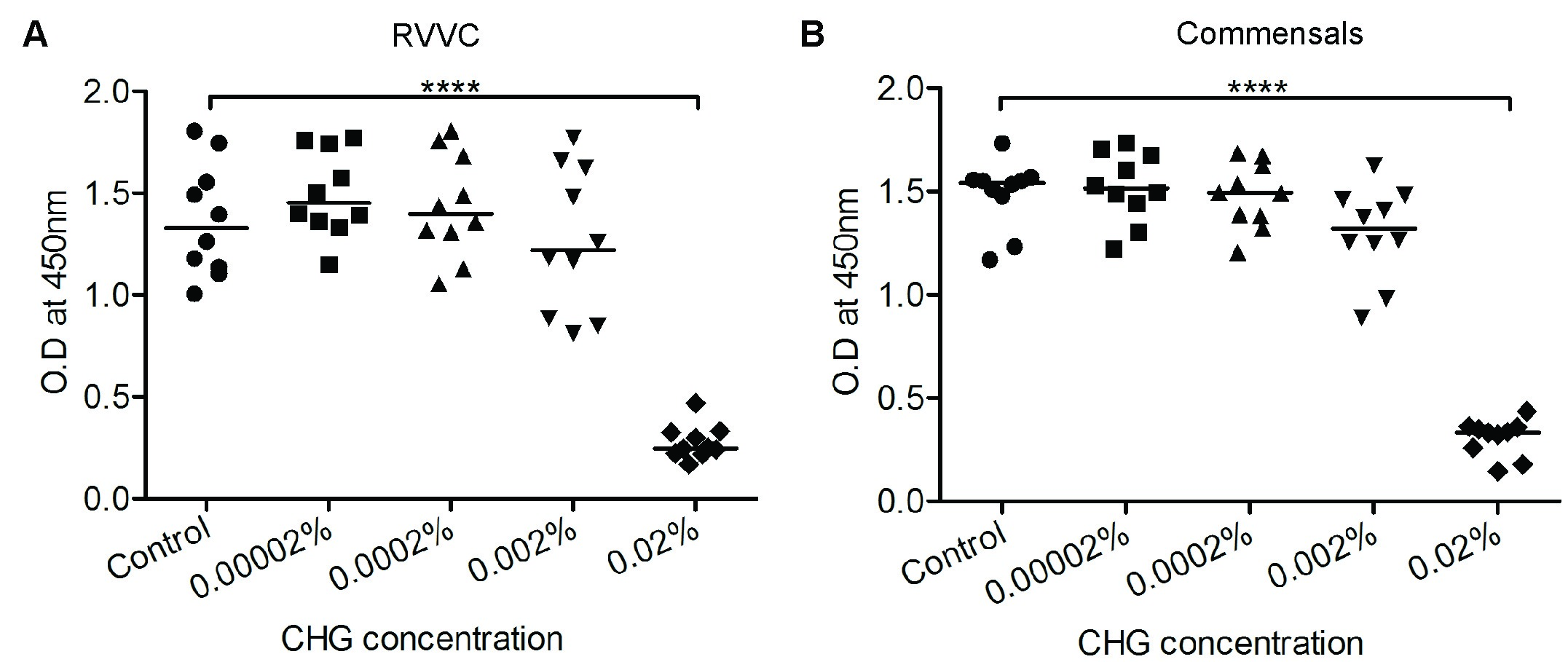

Supplement: S1 Fig — The effect of different concentration of chlorhexidine digluconate (CHG), 0.00002%- 0.02%, were compared to untreated C. albicans was investigated on both RVVC (n = 10) (A) and from asymptomatic women, commensals (n = 10) (B). Metabolic active cells within the mature biofilm, analyzed with XTT assay and measured at 450nm. **** p <0.0001. (TIF) [file pone.0238428.s001.tif]

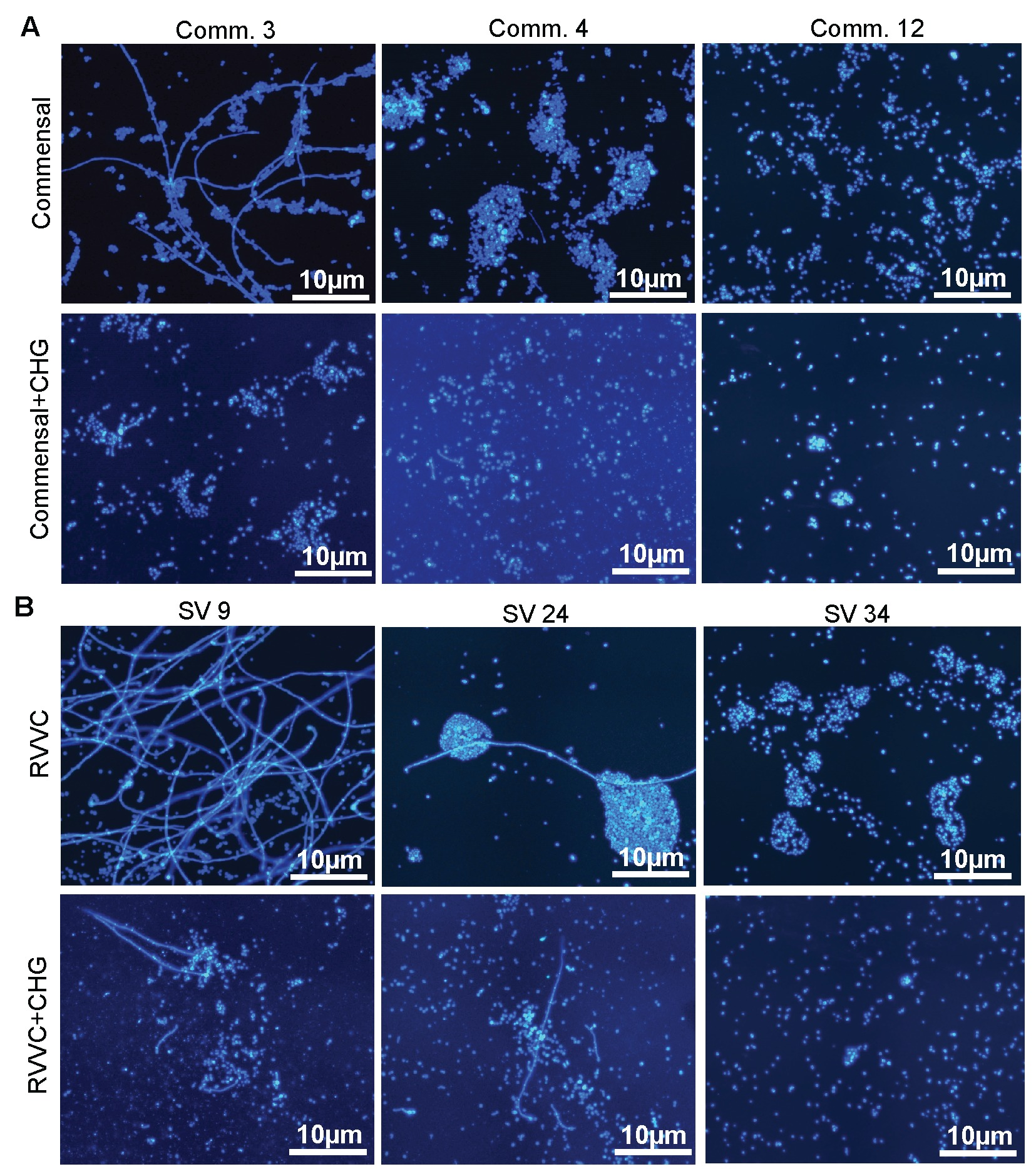

Supplement: S2 Fig — C. albicans obtained from patients with RVVC (n = 10) and from asymptomatic women, commensals (n = 10) were treated with chlorhexidine digluconate 0.02%. Imaging of hyphae from mature biofilm after 48h and another 24h with CHG treatment at 30°C commensals (A) and RVVC (B). Staining was performed using blankophore p, images were captured using 20× objective under UV in Olympus microscope. 3 different representative images are shown. (TIF) [file pone.0238428.s002.tif]
